# Enhancement of Electro-Optical Characteristics in GaN-Based Ultraviolet Laser Diodes Through Upper Optical Confinement Structure Design

**DOI:** 10.3390/nano15080596

**Published:** 2025-04-13

**Authors:** Zhiwei Li, Jing Yang, Lina Yu, Linjun Sun, Min Wu, Weijun Li

**Affiliations:** 1 AnnLab, Institute of Semiconductors, Chinese Academy of Sciences, Beijing 100083, China; zwli@semi.ac.cn (Z.L.); yulina@semi.ac.cn (L.Y.); sunlinjun@semi.ac.cn (L.S.); wumin@semi.ac.cn (M.W.); 2 Center of Materials Science and Optoelectronics Engineering & School of Integrated Circuits, University of Chinese Academy of Sciences, Beijing 100049, China; 3 State Key Laboratory on Integrated Optoelectronics, Institute of Semiconductors, Chinese Academy of Sciences, Beijing 100083, China

**Keywords:** waveguide structure, UV LDs, absorption loss

## Abstract

Two series of laser diodes with different AlGaN cladding layers were investigated, and it was found that the internal absorption loss was reduced by using a u-AlGaN and p-AlGaN composite upper cladding layer, and the performance of LD was improved. The optimized thickness of u-AlGaN is related to the waveguide layer structure of LDs. For LDs with a thick waveguide layer, the u-AlGaN cladding layer should be thinner to achieve high hole concentration and high hole injection efficiency. For LDs with a thin waveguide layer, the u-AlGaN cladding layer can be thick to suppress the internal absorption loss. Thus, a composite upper waveguide structure with a thinner waveguide layer, a thicker u-AlGaN cladding layer, and a p-AlGaN cladding layer is an optimized structure for short waveguide UV LDs due to lower carrier loss and absorption loss.

## 1. Introduction

GaN-based III-V semiconductors can have direct bandgap and a wide bandgap from 0.7 eV to 6.0 eV, and are considered to be the most promising materials for ultraviolet (UV) and visible laser diodes (LDs). GaN-based LDs have the advantage of a wide range of applications such as biochemical analysis, materials processing, laser display, etc. Therefore, the development of GaN-based LDs has been rapid since the 1990s [1,2,3,4,5,6,7]. So far, GaN-based violet, blue, and green LDs have been reported, and GaN-based blue LDs with high output power (5W) have been commercialized [8,9]. Compared to visible LDs, reducing the lasing wavelength below 400 nm, especially 370 nm, faces many challenges due to the large lattice mismatch between the high AlN mole fraction of the AlGaN cladding layer and the GaN substrate. To date, the highest continuous wave (CW) optical output power of a 375–380 nm GaN-based commercially produced LD is approximately 800 mW, which is manufactured by Nichia Corporation. This is much lower than that of blue LDs [10,11,12]. Therefore, research into the realization of the high output power of UV LDs is very important. It is known that the performance of an LD is directly related to its internal absorption loss, and a large internal absorption loss results in a large threshold current density and a low slope efficiency. For GaN-based LDs, the internal absorption loss mainly comes from dislocation scattering, impurity absorption, free carrier absorption, etc., among which the absorption of Mg impurities in p-type materials is an important reason [13,14]. Therefore, the use of asymmetric waveguide structure to make the optical field distribution deviate from the p-type region is the common method to reduce the absorption loss of LDs and has been reported many times previously [15,16]. In addition to the internal absorption loss, carrier loss is also a key factor limiting the characteristics of LDs [15,17]. Increases in the potential barrier of an electron blocking layer (EBL) are usually used in GaN-based visible light emitting diodes (LEDs) or LDs to decrease carrier loss [18]. However, for LDs with UV LDs, it is found that a lot of carriers accumulate and recombine in the WG layer. It is the main reason for the loss of carriers. In this paper, we propose a new composite upper optical confinement structure for UV LDs, which includes a thin upper WG layer, a thick u-AlGaN cladding layer with Al composition graded, and a p-AlGaN cladding layer. A thin upper WG layer is used to suppress the recombination of carriers in the WG layer, but it results in increases in absorption loss due to the increases in optical field distribution in p-AlGaN CL. Therefore, a thicker u-AlGaN cladding layer with polarization doping is used to decrease the absorption of AlGaN CL.

## 2. Laser Structure and Simulation Parameters

**Figure 1:**
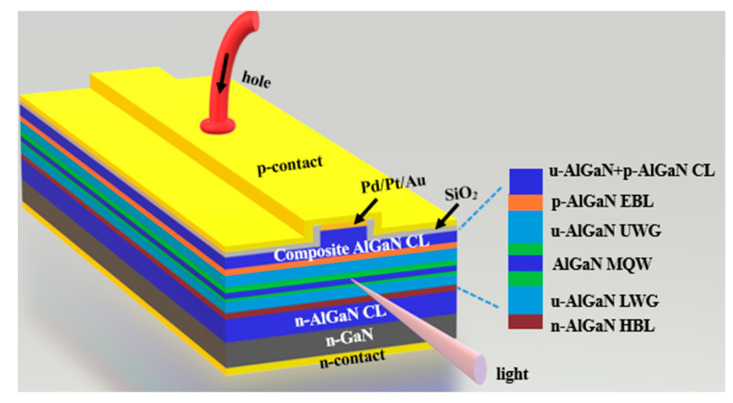
The schematic diagram of the LD structure.

The LD structures used for simulation along the growth direction consist of a 1 μm thick n-type GaN layer, a 500 nm n-type Al_0.07_Ga_0.93_N cladding layer (CL) with an n-doping concentration of 3 × 10^18^ cm^−3^, a 20 nm n-type Al_0.25_Ga_0.75_N hole blocking layer (HBL), an Al_0.03_Ga_0.97_N lower WG (LWG) layer, an unintentionally doped AlGaN/GaN multi-quantum well (MQW) active region with an emission wavelength of about 364 nm, an Al_0.03_Ga_0.97_N upper WG (UWG) layer, a 20 nm p-type Al_0.3_Ga_0.7_N electron blocking layer (EBL), and AlGaN CL. To suppress the carrier loss in the WG layer and absorption for Mg impurities in AlGaN CL, a uniform AlGaN CL have been replaced by a composite AlGaN CL structure, which consists of an undoped AlGaN cladding layer with linear reduction in the AlN mole fraction from 15% to 7% and a p-doped Al_0.07_Ga_0.93_N cladding layer with a Mg doping concentration of 1 × 10^19^ cm^−3^, the total thickness of the composite AlGaN CL structure being maintained at 500 nm. The schematic diagram of these LD structures is shown in Figure 1. In this study, the samples were divided into series I and II according to the thickness of the waveguide layer. For series I, there are six LD samples; the thickness of the WG layer is set to be 150 nm for all the LDs, and the difference for these LDs is the thickness of u-AlGaN CL, which is 0 nm, 20 nm, 50 nm, 100 nm, 200 nm, and 300 nm, respectively, and the LDs are named as LD1, LD2, LD3, LD4, LD5, and LD6, respectively. For series II, there are five samples, and compared with series I, the thickness of the WG layer is set to be a thinner one of 50 nm, the thickness of u-AlGaN CL is 20 nm, 50 nm, 100 nm, 200 nm, and 300 nm, respectively, and the LDs are named as LDII-2, LDII-3, LDII-4, LDII-5, and LDII-6. In this work, the operating characteristics of the LDs were numerically simulated by the LASTIP software(2008.8.29.401), which was developed by Cross light Software Inc. (Vancouver, Canada). LASTIP is a semiconductor LD simulation program to simulate the operation of semiconductor laser diodes in two dimensions (2D), which is simulation by self-consistently solving Poisson’s equation and the current continuity equations by using finite element analysis. The optical model of the laser diodes uses the effective refractive index method and only one lateral mode (the fundamental lateral mode) is considered. The self-heating effect in LDs is not considered in our simulation, and we set the temperature as a constant of 25 °C. The details of the laser model are published elsewhere [19]. For all the LDs, the ridge size was set to 3 μm × 600 μm. The screening factor of polarization was assumed to be 0.25. The absorption coefficients of the u-type, n-type, and p-type layers were set to 10 cm^−1^, 10 cm^−1^, and 50 cm^−1^, respectively. The refractive indices for AlGaN materials were obtained by interpolation method according to those of GaN and AlN [20]. The Mg acceptor activation energy is taken as 170 meV for GaN, which is assumed to increase by 3 meV/% Al for AlGaN [21].

## 3. Results and Discussion

**Figure 2:**
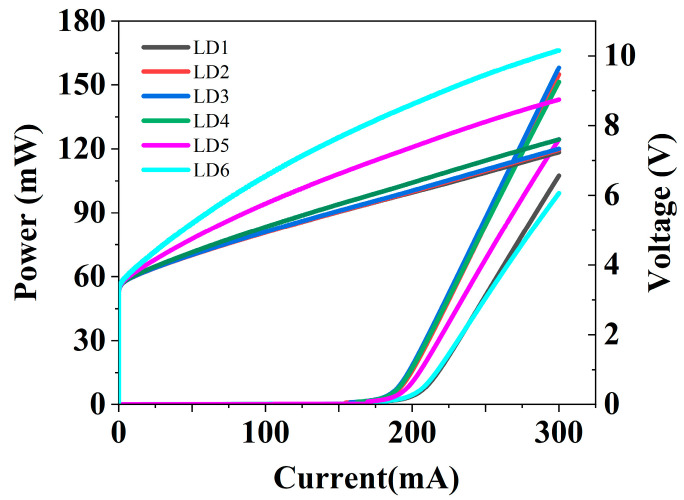
The *P*-*I*-*V* curves of LD1, LD2, LD3, LD4, LD5, and LD6 with the u-AlGaN CL thickness of 0 nm, 20 nm, 50 nm, 100 nm, 200 nm, and 300 nm, respectively, under CW operation.

The *P*-*I*-*V* curves of LD1, LD2, LD3, LD4, LD5, and LD6 under continuous-wave (CW) operation were first simulated and are displayed in Figure 2. As we expected, when uniform p-AlGaN CL (LD1) was changed to composite AlGaN CL (LD2), the threshold current decreased and the slope efficiency increased. This can be attributed to the reduction in absorption loss due to the lower absorption coefficient of u-AlGaN compared to p-AlGaN. However, the performance of the LDs did not improve significantly and then it deteriorated as the thickness of the u-AlGaN CL continued to increase above 50 nm. In addition, it is worth noting that the voltages of LD5 and LD6 were much higher than those of other LDs at all the injection currents, indicating that the series resistance increased as the thickness of the u-AlGaN CL increased to more than 100 nm.

It is well known that the threshold current density $\left(J_{th}\right)$ and the slope efficiency (*SE*) of semiconductor laser diodes can be derived as [22,23,24].

(1) $$J_{th}=J_{t}+\frac{1}{\Gamma A}\left[\alpha_{i}+\frac{1}{L}ln\frac{1}{R}\right]$$

(2) $$SE=\frac{hc}{q\lambda }\frac{\alpha_{m}}{\alpha_{m}+\alpha_{i}}\eta_{inj}$$

where $J_{t}$ is the intercept of the gain curve in the current density coordinate (current density corresponding to the transparent carrier concentration); *Γ* is the optical confinement factor; *α*_*i*_ is the internal absorption loss; *R* is the reflectivity of the cavity surface; *L* is the length of the LD cavity; *h* is the Planck constant; *c* is the speed of light; *q* is the elementary charge; *λ* is the emission wavelength; *α_m_* is the mirror loss; and $\eta_{inj}$ is the carrier injection efficiency, which is the ratio of carriers injection into the active region to the total carriers. Thus, reducing internal absorption loss should lead to a reduction in the threshold current and an increase in *SE* according to Equations (1) and (2).

**Figure 3:**
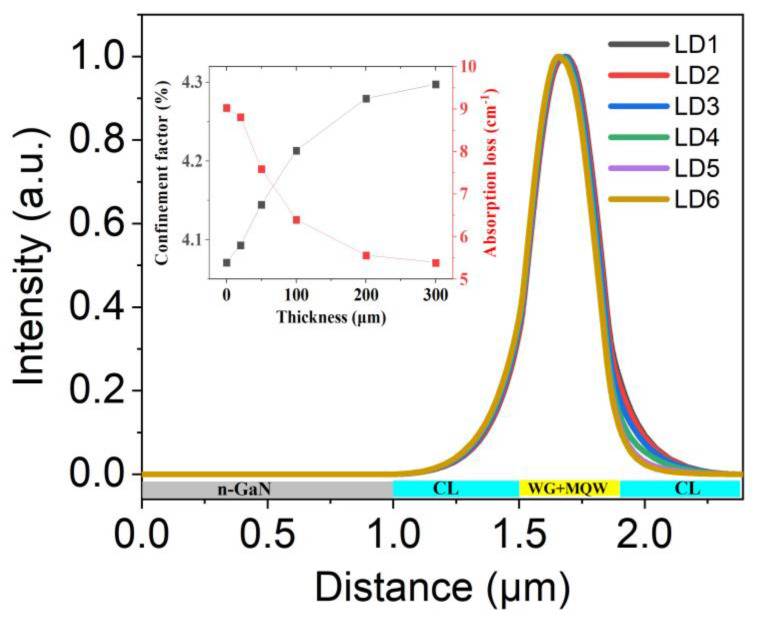
Optical field distribution at the center of LD along the growth direction for LD1, LD2, LD3, LD4, LD5, and LD6; the inset shows the dependence of optical loss and optical confinement factor on u-AlGaN CL thickness.

Since the absorption loss in GaN-based LDs is largely dependent on the absorption of Mg impurities in the p-AlGaN layer [13,14], the absorption loss should decrease as the increase in the thickness u-AlGaN CL, in which case the performance of LD should be improved. It is not consistent with our simulation when the thickness of u-AlGaN CL increases to more than 50 nm. Therefore, the reason for the decrease in LD performance should be investigated. We first checked the optical field distribution of LDs, which have been shown in Figure 3. The confinement factor and absorption loss were calculated from the optical field distribution. The data are shown in the inset of Figure 3. As we expected, the confinement factor increased, and the absorption loss decreased with increasing u-AlGaN CL thickness from 0 to 300 μm. It indicates that other factors may lead to the deterioration of the LD characteristics as the u-AlGaN CL becomes thicker.

Equation (2) shows that the *SE* is related to the mirror loss and the injection efficiency, except for the internal absorption loss. The mirror loss is related to the reflectivity of the cavity surface and has been set to a constant. Therefore, the injection efficiency can be different for these LDs.

**Figure 4:**
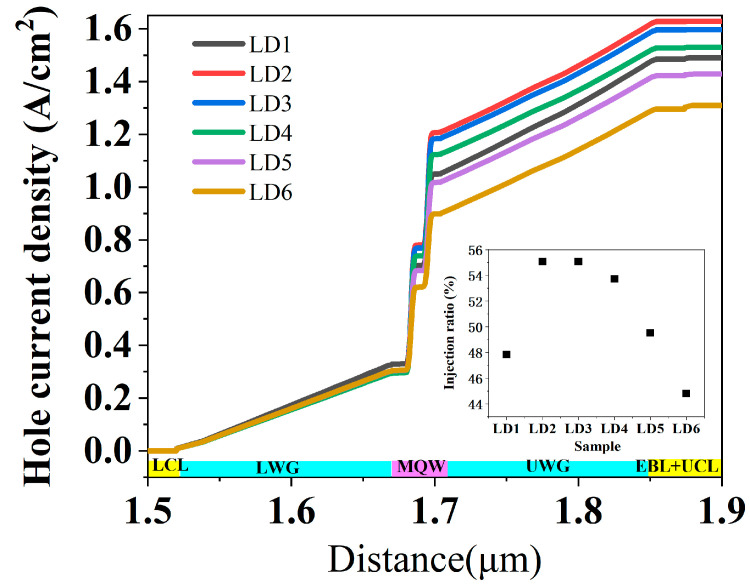
Spatial distribution of hole current density at the center of LD along the growth direction for LD1, LD2, LD3, LD4, LD5, and LD6; the inset shows the dependence of injection ratio of holes on u-AlGaN CL thickness.

In fact, the AlGaN CL composite structure is not only a cladding layer that confines the optical field, but also a hole providing layer. The hole concentration in this layer is related to the carrier injection efficiency and has an important effect on the performance of the LDs. The spatial distribution of hole current density at an injection current of 300 mA for LD1, LD2, LD3, LD4, LD5, and LD6 were obtained and are shown in Figure 4. The hole current is mainly due to hole drift under the influence of an electric field or diffusion under a concentration gradient. A high hole current density in the p-type layer means that more holes are transported from this layer, and then these holes are injected into the MQW region and recombined with electrons in quantum wells, resulting in a reduced hole current density in the quantum well profile [20]. It suggests that the reduction in hole current density in quantum wells is directly related to hole recombination. Thus, it is proportional to the injected carriers, since the injected carriers are used for stimulated emission after lasing. In this paper, the ratio between the hole current density reduction in QW layers and the hole current density in p-AlGaN CL was used to represent the hole injection efficiency of LDs when the injection current was higher than the threshold current. The hole injection ratio of all the LDs under the injection current of 300 mA was calculated and is shown in the inset of Figure 4. It increased from 47.8% to 55.07% when the uniform AlGaN was changed to composite AlGaN CL, and then it decreased to 44.8% when the thickness of the u-AlGaN layer increased from 20 nm to 300 nm. It indicates that the hole injection into the MQW region decreases as the thickness of u-AlGaN CL increases. It is attributed to the fact that the polarization charge density and hole concentration decrease in thick u-AlGaN CL. It agrees well with the hole current density in the upper cladding layer shown in Figure 4 and the voltages shown in Figure 2. The lower hole injection efficiency is the reason for the deterioration of the LD with thicker u-AlGaN. In addition, we found that LD2 and LD3 with thinner thickness u-AlGaN CL had both lower absorption loss and higher hole injection efficiency compared to uniform AlGaN CL, and a thin gradient u-AlGaN CL was equivalent to a hole injection layer and the performance of LDs with a thin gradient u-AlGaN CL could be improved.

**Figure 5:**
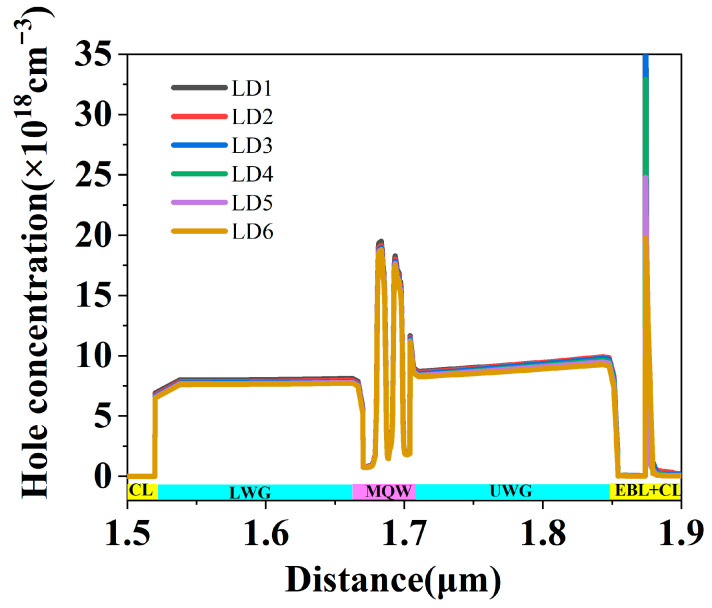
Spatial distribution of the hole concentration at the center of LD along the growth direction under the injection current of 300 mA.

In fact, it should be noted that there was a large reduction in hole current density in the UWG layer and LWG layers, as shown in Figure 4, which is attributed to the high concentration of carriers in the WG layer, as shown in Figure 5. These carriers do not contribute to the laser and are detrimental to the LDs. However, since the tensile strain in the AlGaN layer increased with increasing AlN mole fraction when grown on free-standing GaN substrates [25], the highest AlN mole fraction of AlGaN CL was about 7–8%, and the Al content of the AlGaN WG layer was only 5% and even low. This results in a low energy barrier between the WG layer and the MQW region in UV LDs, especially for LDs with shorter lasing wavelengths. In this case, the carrier confinement effect became weak and many carriers were consumed in the WG layers. This led to a higher threshold current for UV LDs, which is also the main difficulty for short-wavelength UV LDs.

**Figure 6:**
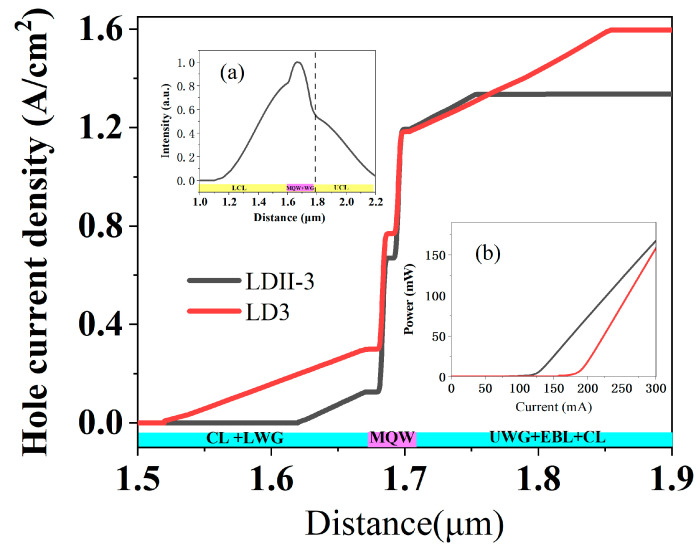
Spatial distribution of hole current density of LDII-3 and LD3; the inset (**a**) shows the optical field distribution at the center of LD II-3 (**a**) along the growth direction, and the region at the right of the dotted line in the inset (**a**) is the CL region. The inset (**b**) shows the *P*-*I* curves of LDII-3 and LD3.

To reduce the carrier loss in the WG layer, LDs with a thinner WG layer, LDII-3, were simulated. Compared to LD3, its LWG and UWG layers were reduced from 150 nm to 50 nm. The spatial distribution of hole current density, P-I curves, and optical field distribution are shown in Figure 6. The hole loss in the WG layer decreased and the hole injection ratio obviously increased from 55% to 79.5%, and the threshold current decreased from 175 mA to 118 mA. However, due to the thin WG layer, a larger proportion of the optical field penetrated into the CL, as shown in the inset (a) of Figure 6. It will lead to large absorption loss because the optical field is distributed in the p-AlGaN CL. In this case, composite AlGaN CL with thick u-AlGaN thickness may be suitable.

**Figure 7:**
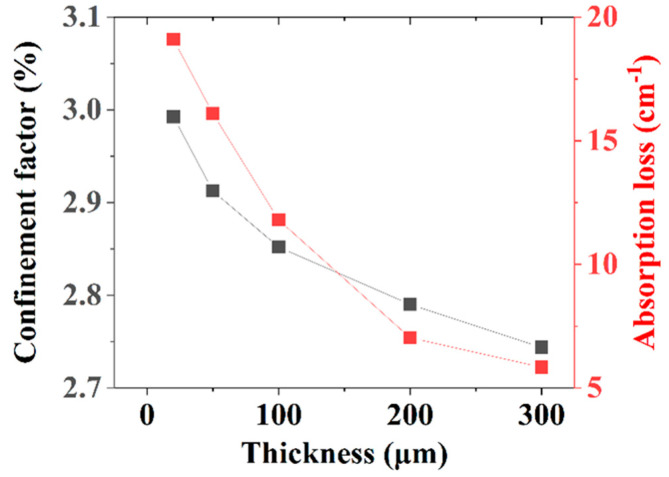
Dependence of optical loss and optical confinement factor on u-AlGaN CL thickness for series II.

**Figure 8:**
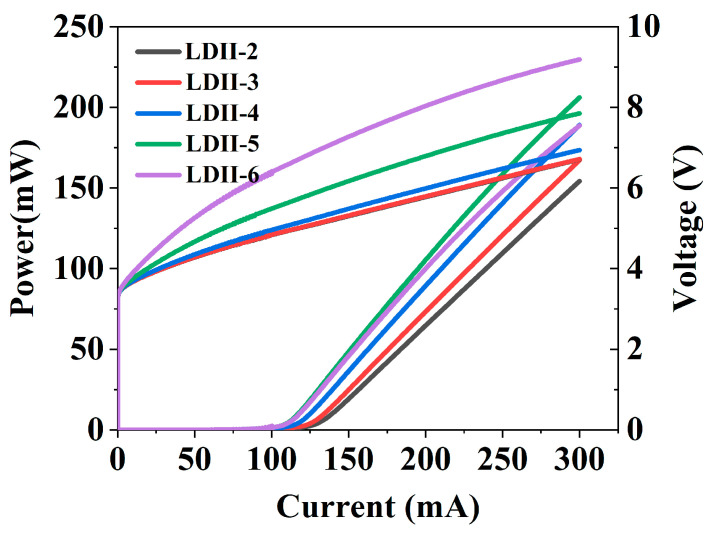
*P*-*I*-*V* curves under CW operation of LDs with different thicknesses of the u-AlGaN layer for series II.

LDs for series II with a WG thickness of 50 nm were simulated, and the optical confinement factor and absorption loss were obtained and are shown in Figure 7. It can be seen that the absorption loss obviously decreased from nearly 19.1 cm^−1^ to 5.8 cm^−1^, and the variation was much larger than that of series I. Therefore, the *P*-*I* curves were obviously improved when the thickness increased from 20 to 200 nm as shown in Figure 8, even though the hole injection efficiency decreased (it can be seen from the voltages of LDII-5 and LDII-6). The composite upper optical confinement structure including a thin WG layer, a thick u-AlGaN, and a p-AlGaN CL is advantageous for improving the performance of UV LDs with short laser wavelengths where carrier loss is obvious.

## 4. Conclusions

Two series of GaN-based UV LDs with different AlGaN cladding layers were investigated. It was found that the threshold current decreases and the slope efficiency increases when the thin u-AlGaN is partial instead of p-AlGaN as the cladding layer. It is attributed that a thin u-AlGaN layer not only provides a high hole concentration, but also reduces the absorption loss due to polarization doping. However, due to the reduction in hole concentration, the performance of LDs deteriorates as the u-AlGaN thickness further increases. In addition, we also found that the optimized thickness of u-AlGaN is related to the waveguide layer structure of the LDs. For LDs with a thin waveguide layer, the u-AlGaN cladding layer can be thick to suppress the internal absorption loss even though the hole concentration decreases. Therefore, a composite upper optical waveguide structure with a thinner waveguide layer, a thicker u-AlGaN CL, and a p-AlGaN cladding layer is an optimized structure for short waveguide ultraviolet LDs due to lower carrier loss and absorption loss.

## Data Availability

The data presented in this study are available upon request from the corresponding author upon reasonable request.
